# Evaluation of the Upper Arch Morphological Changes after Two Different Protocols of Expansion in Early Mixed Dentition: Rapid Maxillary Expansion and Invisalign^®^ First System

**DOI:** 10.3390/life12091323

**Published:** 2022-08-26

**Authors:** Elisabetta Cretella Lombardo, Valeria Paoloni, Silvia Fanelli, Chiara Pavoni, Francesca Gazzani, Paola Cozza

**Affiliations:** 1 Department of Systems Medicine, University of Rome “Tor Vergata”, Viale Oxford 81, 00133 Rome, Italy; 2 Department of Dentistry, Universiteti Katolik “Zoja e Këshillit të Mirë”, 1026 Tirana, Albania; 3 Department of Health Sciences, UniCamillus-Saint Camillus International University, 00133 Rome, Italy

**Keywords:** maxillary expansion, RME, Invisalign^®^ First system, geometric morphometrics

## Abstract

**Background:** The objective of this retrospective study was to analyze the morphological changes of the upper arch after two protocols of expansion, the Invisalign® First system and rapid maxillary expansion (RME), in mixed dentition by means of geometric morphometric analysis (GMM). **Methods:** Digital dental casts of 32 children treated either with RME (RME group: 17 subjects; mean age 8.1 years) or the First system (First group: subjects; mean age 8.4 years) were collected. For both the RG and FG, pre-(T1) and post-treatment(T2) digital models were created. A total of 14 landmarks were digitized and GMM was applied. Procrustes analysis and principal component analysis (PCA) were performed. **Results:** The PC1 resulting from the T2–T1 comparison in the RG g showed statistically significant morphological changes in the posterior region of the upper arch shape, without significant variations in the anterior region. The comparison of the T2–T1 changes in the FG showed an increase in the transverse dimension at the level of the canine and the first deciduous molar widths, with morphological variation in the anterior region due to frontal teeth alignment. **Conclusions:** The First system induced shape modifications of the upper arch during expansion in contrast to RME. The FG presented an improvement in the maxillary arch shape, while the RG maintained the initial triangular shape.

## 1. Introduction

Maxillary expansion treatments have been used for more than a century to correct maxillary transverse deficiency, and various treatment approaches can be found in the literature [1,2,3,4]. Among all, rapid maxillary expansion (RME) is the most effective orthopedic procedure to increase the maxillary transverse dimension in young patients by opening the midpalatal suture, which has not yet completely ossified in growing individuals [5,6,7,8,9,10,11,12,13]. As reported by Paoloni V. et al., RME induces a long-term improvement of the upper intercanine and intermolar width, with an anterior overall gain of 2.9 mm and posterior overall gain of 4.4 mm [1]. The aim of rapid maxillary expansion is to create heavy forces at the sutural site over a short period of time, and it produces midpalatal suture separation by the disruption of the sutural connective tissue. Forces produced by the RME appliance have been reported in the range of 16–20 kg [1].

Maxillary constriction can be associated with several problems that include occlusal disharmony, aesthetics, and functional difficulties, such as the narrowing of the pharyngeal airway, increased nasal resistance, alterations in the tongue posture, and mouth breathing. Therefore, early treatment of this malocclusion through palatal expansion is strongly recommended [1].

However, in recent years clear aligners have been proposed as a more comfortable alternative to conventional approaches to obtain dento-alveolar maxillary expansion in growing subjects. Among the latest innovations, Align Technology (Santa Clara, CA, USA) introduced the Invisalign^®^ First system, consisting of clear aligners for patients between the ages of 6 and 10 years, to perform phase I of orthodontic treatment, including the correction of a narrow maxillary arch [14,15,16,17,18,19,20,21,22,23].

The possibility of combining dentoalveolar expansion, teeth alignment, and the recovery of the correct arch form at the same time, thus reducing treatment times, represents the main advantage of this device compared to standard devices.

Technological advances in diagnostic tools have simplified the visualization of morphological shape changes after orthodontic treatment. Among them, the geometric morphometrics method (GMM) is a special method applied to understand shape variation, especially in 3D, where the shape complexity is at its maximum [24,25,26,27,28].

In the literature, only a few recent studies have evaluated the transverse maxillary arch development with the Invisalign^®^ First system in growing subjects by means of transversal linear and angular measurements on digital casts [14,15,18].

Levrini et al., in 2021, evaluated the dentoalveolar changes in 20 patients treated with Invisalign^®^ First in the mixed dentition, reporting a significant increase in all the measurements regarding the arch width.

A further study was published by Lione et al. in 2021. In this prospective investigation, transverse interdental widths were measured in the upper arch on each digital model at the beginning and at the end of the Invisalign^®^ First system treatment. The authors concluded that the greatest increase in the maxillary width was detected at the level of the upper first deciduous molars, followed by expansion at the level of the second deciduous molars and expansion at the level of the deciduous canines. The upper first molars showed greater expansion in the intermolar mesial width due to the rotation that occurred during the expansion around the palatal root of the tooth.

However, to our knowledge, no data are available regarding the morphological changes of the maxilla in a growing subject treated with clear aligners.

In the current investigation, the GMM method was used to address an increasingly varied range of questions about maxillary expansion with different protocols.

The aim of this retrospective study was to analyze the morphological changes of the upper arch using two different protocols of expansion (First system and RME) in mixed dentition by means of geometric morphometric analysis (GMM).

## 2. Materials and Methods

**Figure 1:**
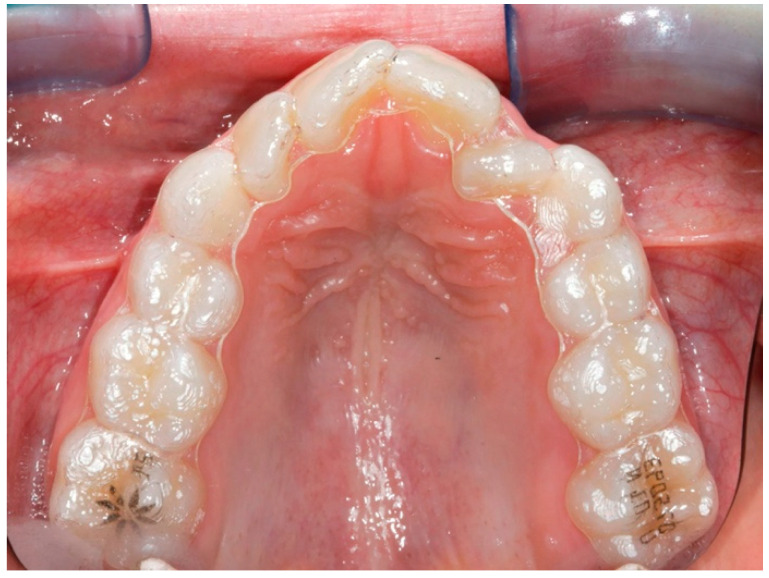
Invisalign^®^ First clear aligners.

**Figure 2:**
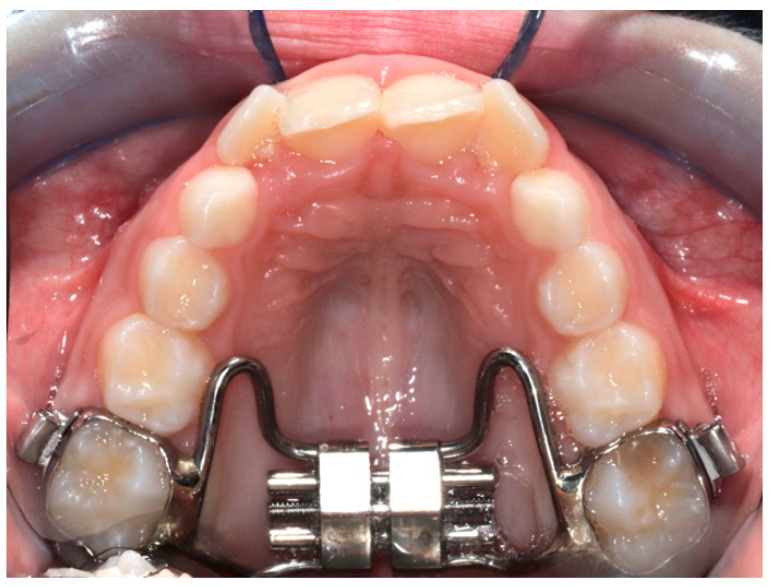
Conventional RME.

The digital dental casts of 32 children consecutively treated with either RME (Figure 1) (RG: n = 17, 8 males, 9 females; mean age 8.1 ± 0.8 years) or the Invisalign^®^ First system (Figure 2) (FG: n = 15, 7 males, 8 females; mean age 8.4 ± 1.1 years) were collected. The study subjects were retrieved from the records of patients treated at the Department of Orthodontics at the University of Rome “Tor Vergata”.

To be included in the study, patients had to present with the following characteristics: European ancestry, posterior transverse discrepancy between the maxillary and mandibular arches up to 6 mm, mixed dentition stage, the presence of first permanent molars, and a high level of compliance.

The posterior transverse inter-arch discrepancy was obtained by calculating the difference between the maxillary intermolar width (distance between the central fossae of the right and left first maxillary molars) and the mandibular intermolar width (distance between the mesiovestibular cusps of the right and left first mandibular molars).

The level of compliance was assessed with a face-to-face interview conducted by a single investigator using a 3-point Likert-type scale (poor, moderate, and high) at the end of the treatment: poor compliance was declared when the patient wore the appliance at night only, moderate compliance happened when the patient wore the appliance at night and during the day at home, and high compliance was established when the patient wore the appliance full time, as suggested by the clinician.

The exclusion criteria included multiple and/or advanced caries, tooth agenesis, supernumerary teeth, cleft lip and/or palate, and periodontal diseases. None of the patients had any oral habits to require myofunctional treatment or muscle re-education. All patients presented a mesial step or a flush terminal plane molar relationship.

This project was approved by the ethical committee at the University of Rome “Tor Vergata” (protocol number 163.20) and informed consent was obtained from the patients’ parents.

The First group (FG subjects underwent a non-extraction treatment protocol with Invisalign^®^ First clear aligners with no auxiliaries other than attachments and no enamel interproximal reduction (IPR).

The ClinCheck^®^ for each patient of FG was planned with the same standardized expansion protocol: sequential staging pattern for upper arch expansion, “molars move first”, followed by the simultaneous expansion of posterior deciduous teeth and canines. According to Lione et al. [18], the amount of arch expansion was 0.15 mm per stage [18]. For the upper first molars, a simultaneous distorotation according to Rickett′s line [29] and 2 degrees of extra buccal root torque were required for each phase of expansion. The overcorrection of the transverse upper dimension was never prescribed, but a cusp–fossa relationship was digitally planned.

All patients of FG were instructed to wear their aligners full-time. The patients changed the aligners every 7 days, and every 4 stages, the clinician checked the good aligner fit. Optimized attachments were placed on the basis of the tooth surface using software. The treatment lasted 8 months, and digital scans were taken 3 months after the end of the therapy to create digital dental casts.

The RME group (RG) subjects were treated with a butterfly palatal expander (expansion screw with telescopic guides, A2620—Leone SpA, Sesto Fiorentino, Firenze, Italy) [30]. This appliance has a butterfly-shaped stainless-steel framework banded on the first maxillary molars that extend forward to the palatal surfaces of the maxillary deciduous molars, and the activation of the screw commenced immediately after the appliance was cemented in place.

In the RG, the RME expansion screw was activated by the patients’ parents at a 1/4 turn per day (one activation, 0.25 mm per turn) until overcorrection was achieved (i.e., the palatal cusps of the maxillary posterior teeth approximated the buccal cusps of the mandibular posterior teeth). The RME was kept in place on the teeth as a passive retainer stabilizing the expansion reached during the screw activation and was removed 8 months after the application. During the active phase of treatment, the patients were checked every 2 weeks to monitor the activation of the screw. In addition, in this group of patients, the average treatment time was 8 months, and intra-oral scans were taken 3 months after the end of active therapy to create digital dental casts.

In both groups, no removable or cemented retention appliance was applied after the end of active therapy. For both the RG and FG, pre-treatment (T1) and post-treatment (T2) digital models (.stl files) were created using iTero scans. All models were exported in a .stl digital file and uploaded to specific software, Viewbox 4.0 (dHAL Software, Kifissia, Greece), in order to digitize the casts.

Fourteen landmarks for maxillary dentition were digitized on the digital dental casts using the specific software Viewbox 4.0 dHAL Software, Kifissia, Greece) for customized digitization. All measurements were calculated at T1 and T2 by a single operator (S.F.) and then checked by a second operator (E.C.L.). All the reference points were verified; any differences were resolved by retracing the reference points to satisfy both operators.

**Figure 3:**
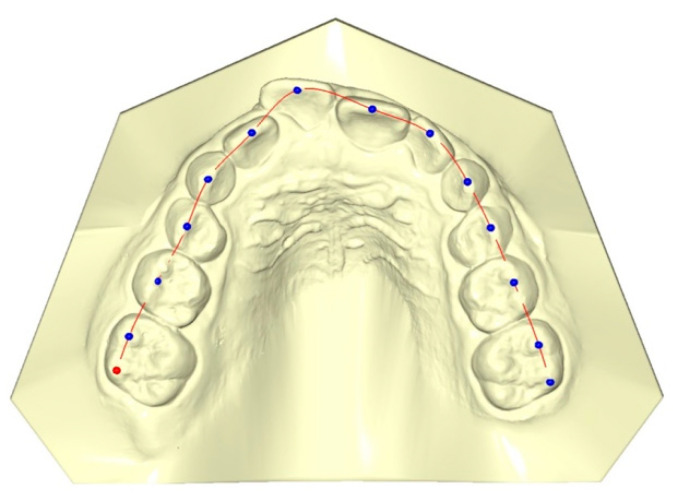
Digitized landmarks on maxillary arch.

The landmarks chosen for digitization were the middle of the incisal edge of the central and lateral incisors, the cusp tips of deciduous canines and the first deciduous molars, the sulcus of the second deciduous molars, and the vestibular cusps of the first molars (Figure 3). Each set of landmarks represents the dental arch form of the upper jaw in three dimensions. A perimeter curve passing through the landmarks joins them together (Figure 3).

### Statistical Analysis

To determine the reliability of the method, 20 maxillary dental casts were randomly selected and re-digitized by the same operators 10 days after the first session. The random error was expressed as the distance between repeated digitizations in the shape space compared to the total variance of the sample.

To test the intra- and inter-operator differences, intra-class correlation coefficients (ICCs) were used to check the reliability of the first and second measurements.

Procrustes analysis was applied, and principal component analysis (PCA) was performed to reveal the main patterns of the dental shape variation in the intra-group comparisons (T2–T1 in RG and T2–T1 in FG). The analysis was conducted with Viewbox 4.0 (dHAL Software, Kifissia, Greece) (PCA).

The test used to evaluate the statistical differences between the two groups was the Procrustes distance between the means, with 10,000 permutations.

## 3. Results

The mean random error of the 20 repeated digitizations, expressed as a percentage of the total shape variance of the sample, was 2.7% (range: 1.01–4.82%, SD = 0.84%).

The ICC values show excellent agreement for both the intra- and inter-observer reliability, ranging from 0.993 to 0.997.

**Figure 4:**
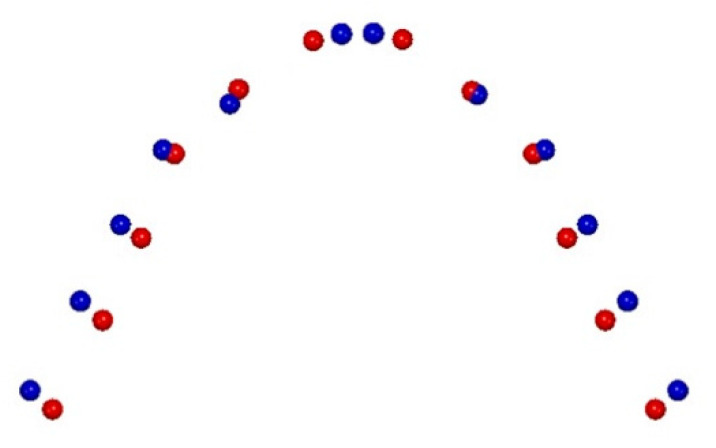
Graphic depiction of upper arch morphological shape changes in the RME group (T2—blue points, T1—red points).

From the comparison of the T2–T1 changes in the RG (Figure 4), the first four principal components (PCs) are statistically meaningful (at least 5% of the total shape variability), and comprise 82.8% of the total shape variability (PC1 = 62.7%, PC2 = 8.3%, PC3 = 6.7%, PC4 = 5.1%).

The variability described by the first PC1 is morphologically the most significant because it defined 62.7% of the total shape variability.

The PC1 resulting from the comparison of T2–T1 in the RG shows statistically significant morphological changes in the posterior region of the upper arch shape at the level of the first permanent molars, without significant variations in the anterior region (10,000 permutations; *p* = 0.042).

**Figure 5:**
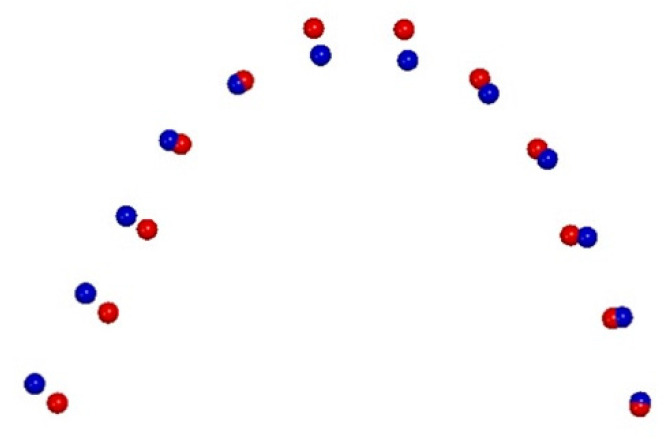
Graphic depiction of upper arch morphological shape changes in the First group (T2—blue points, T1—red points).

From the comparison of the T2–T1 changes in the FG (Figure 5), the first three principal components (PCs) are statistically meaningful (at least 5% of the total shape variability) and comprise 78.5% of the total shape variability (PC1 = 63.5%, PC2 = 8.3%, PC3 = 6.7%).

The comparison of the T2–T1 changes in the FG shows an increase in the transverse dimension in the lateral segments, specifically at the level of the canine and the first deciduous molar widths. At the same time, a morphological variation can be observed in the anterior region due to the alignment of frontal teeth clear on a more physiological anterior dental arch curve (10,000 permutations; *p* = 0.038).

## 4. Discussion

Maxillary expansion is one of the treatment options for the correction of the skeletal constriction of the upper jaw (e.g., posterior crossbite, anterior crowding, crowding, or arch length discrepancy due to narrow arches), with the intent to increase the transverse widths of the maxilla through the opening of the midpalatal suture. Mid-palatal suture opening can be accomplished in both children and adults, but with advancing maturity, the rigidity of the skeletal components limits the extent and stability of the expansion, which may involve fracturing the bony interdigitations [31].

As reported by Bucci R. et al. (2016), the expansion of the maxilla can be achieved by means of different expansion rates and forces and with different appliances, and the choice among these options can influence the resulting effects of the treatment and the relative relapse [31]. Several appliances and different forces (e.g., rapid maxillary expansion—RME, slow maxillary expansion—SME) exist for the treatment of an upper transverse deficiency, and among these, one of the most common is rapid maxillary expansion [1,2,3,4,5,6,8,9,31]. Numerous investigations into rapid maxillary expansion (RME) have been undertaken in the last 150 years [1,2,3,4,5,6,7,8,9,10,11,12,13]. At present, it is widely accepted that RME causes an opening of the midpalatal suture through the use of forces of a large magnitude, and it produces observable changes in the maxillofacial skeleton [6].

These forces decrease slowly during the 5–7 weeks retention phase, and basal bones continue to relapse until 10 months after the expansion [32]. The use of RME is recommended before the pubertal growth spurt, because ossification of the suture increases thereafter, and the time required to remineralize and regenerate is longer after the expansion process [33].

Because of the various positive side effects on the patient′s general health, the number of indications for RME has grown dramatically over the years.

However, in recent years, Align Technology introduced Invisalign^®^ First as an innovative orthodontic appliance that can be used to correct issues with arch development, expansion, and tooth crowding [14,15,18,19,21,22].

The purpose of this study was to evaluate the morphological maxillary arch changes achievable with clear aligners, comparing the Invisalign^®^ First system with traditional RME treatment by means of geometric morphometric analysis.

Our results show statistically significant morphological changes in the upper arch shape with an increase in the transverse dimension of FG subjects in the anterior region at the level of inter-canine and first inter-deciduous molar widths when compared to the RG subjects (Figure 4 and Figure 5).

This could be explained by the aligner structure, which contains and covers entirely the clinical crown of the teeth. Moreover, digital planning with clear aligners allows for modifying the upper arch form at all levels simultaneously and starting an earlier alignment of the frontal teeth. This can be achieved as the clear aligner acts as a single working unit, and the action of posterior expansion is accompanied by a reaction anteriorly. This condition is not possible with the treatment performed with butterfly RME because the clinical crown is uncovered and free, and the treatment dental effects are limited to the anchoring teeth.

Similar observations were reported in the study conducted by Levrini et al. (2021) [14] about maxillary arch changes in patients treated with the Invisalign^®^ First system. The authors reported a significant increase in all the measurements regarding the arch width. In this retrospective study, the superimpositions of the pre- and post-treatment digital dental models revealed that clear aligners could be a reasonable alternative to a traditional slow maxillary expander in the cases of mild crowding or limited transverse deficiency. The authors found significant increases in all measurements regarding arch width and arch perimeter (+0.85 ± 1.63 mm), while arch depth (−1.24 ± 1.06 mm) and molar inclination (−4.62 ± 6.61 mm) significantly decreased.

In addition, Lione et al. (2021) [18] also confirmed our results showing that Invisalign^®^ First can be considered effective in growing patients who require maxillary arch development.

Invisalign^®^ First allows for expanding a narrow maxilla, changing the arch form and resulting in esthetic and functional improvement. In the cited study [18], the authors compared the changes in the transverse maxillary arch dimensions obtained by means of the Invisalign^®^ First system with a specific expansion protocol in subjects in the early mixed dentition. The expansion protocol chosen for all subjects was “molars move first”, and the predetermination of the maxillary arch form was digitally planned before treatment. The authors reported that at the end of treatment (T2), the greatest net increase was detected at the level of the upper first deciduous molars (+3.7 ± 1.4 mm; *p* < 0.001), followed by the second deciduous molars (+3.4 ± 1.6 mm; *p* < 0.001) and the deciduous canine (+2.6 ± 2.0 mm; *p* < 0.001) [18]. Furthermore, as regards the percentage of the effective transversal expansion (T2) in relation to the planned one with the Clincheck (T2 Clincheck), the first molar showed the highest predictability (83%), followed by the deciduous canine (81%), the second deciduous molars (79%), and the first deciduous molars (77%).

However, neither of the above studies performed a morphological analysis of the maxillary shape modification but only a bidimensional conventional analysis of the Invisalign^®^ First effects in a group of patients in mixed dentition, without considering other treatment approaches.

In the literature, only one study performed by Deregibus et al. in 2020 [34] evaluated the morphological changes of the upper arch in a group of patients treated with Invisalign^®^. The authors selected a total of 27 class II patients with a maximum of 4 mm of expansion planned (maximum 2 mm per hemi-arch) during Invisalign^®^ treatment. The patients’ digital dental casts at T0 (pre-treatment), T1 (accepted set-up) and T2 (retention phase) were compared in order to obtain data regarding the differences between the first prescribed virtual result and the final clinical result in terms of the arch shape.

At T1, the authors reported the presence of wider maxillary and mandibular dental arches compared to T0, with maximum movements observed in the premolar regions (maximum movement 1.94 mm for tooth 15; *p* < 0.0001). At the T1-T2 comparison, more buccal positions of tooth 22, tooth 23, and tooth 24 (maximum movement 0.56mm; *p* < 0.05) were observed. More lingual positions of tooth 37 (maximum movement 0.81 mm; *p* < 0.01), tooth 36, and tooth 47 were observed at the same time (T1-T2).

The authors concluded that the Invisalign^®^ treatment resulted in a significant increase in the arch width at the molar and premolar levels in both arches, according to the prescription. Moreover, the authors stated that orthodontic treatment with Invisalign^®^ might produce functional and stable outcomes, and the differences between the planned and achieved tooth positions, even if statistically relevant, were not deemed clinically important. However, the cited paper analyzed a group of class II patients in complete permanent dentition.

To our knowledge, our study is the only investigation evaluating the morphological changes to the maxillary arch after Invisalign^®^ and RME treatment in a group of patients in mixed dentition.

The two types of treatment are deeply different from each other; the rapid maxillary expander is an orthopedic appliance that aims to reach skeletal effects rather than dental, whereas clear aligners act by pushing on the clinical dental crown, inducing dento-alveolar changes. Regardless of the common knowledge about these differences, we decided to compare them to evaluate the strengths of each and to understand when one appliance is more appropriate than the other in mixed dentition. The choice to band the RME on the first maxillary molars was made to make homogenous the two study samples, even if the second deciduous molars were suitable for the anchorage. We discussed these aspects in the discussion section.

The limitations of the present investigation are its short-term nature and the small sample size of the treated groups. Therefore, further evaluations are necessary to increase the sample size and to analyze the stability of the results obtained in the long term.

## 5. Conclusions

The Invisalign^®^ First treatment can induce significant morphological modifications of the upper arch shape compared to RME therapy.

At the end of the treatment, the FG subjects presented an improvement in the maxillary arch shape differently from the RG subjects, who maintained the initial triangular shape.

## Data Availability

The datasets used and/or analyzed during the current study are available from the corresponding author upon reasonable request.
